# Post-stroke aphasia rehabilitation using computer-based Arabic software program: a randomized controlled trial

**DOI:** 10.1186/s43163-021-00144-3

**Published:** 2021-07-28

**Authors:** Engy Samy Elhakeem, Sabah Saeed Gommaa Mohamed Saeed, Ramy Nabil Abd-Elkader Elsalakawy, Reham Mohamed Elmaghraby, Ghada Abdel Hady Ossman Ashmawy

**Affiliations:** 1 Department of Otorhinolaryngology, Unit of Phoniatrics, Faculty of Medicine, University of Alexandria, 8 Fahmy Wisa Street, Keroseiz Building D, Louran, Alexandria, Egypt; 2 Department of Otorhinolaryngology, Unit of Phoniatrics, Faculty of Medicine, University of Alexandria, 128 Mehwar Elmahmodia, Madinat Elshabab building C, Meharm Bek, Alexandria, Egypt; 3 Rawag Systems Company, 8 Elgretly Street, Camp Ceasar, Alexandria, Egypt; 4 Department of Otorhinolaryngology, Unit of Phoniatrics, Faculty of Medicine, University of Alexandria, 12 Elnahda Street, Roushdy, Alexandria, Egypt; 5 Department of Neuropsychiatry, Faculty of Medicine, University of Alexandria, 158 Por Saeed Street, Elabrahemia, Alexandria, Egypt

**Keywords:** Aphasia, Stroke, Language therapy, Computer therapy, Software

## Abstract

**Background:**

Aphasia is considered an acquired communication disorder. Language intervention in aphasia enhances the patient outcomes. Recently, computer programs are developed for the treatment of aphasia. It is an effective and a low-cost therapy choice. The aim of the study was to assess the effectiveness of language therapy using a computer-based Arabic software program for rehabilitation of post-stroke Arabic-speaking aphasic patients in comparison to the conventional language therapy. We conducted a randomized controlled trial with blinded endpoint evaluation. The trial included 50 aphasic patients. They were randomized into either group I or group II to receive 48 therapy sessions using the Arabic software program (group I) or the conventional therapy (group II). The primary outcome was a measure of improvement in language abilities. It was measured using the Arabic version of the Boston Diagnostic Aphasia Examination to detect any significant improvement in the language of both groups in comparison to pre-therapy results. The post-therapy results of both groups were compared to each other to document the effectiveness of the software program.

**Results:**

A total of 105 aphasic patients were screened and 50 subjects were randomized to the intervention groups [40 subjects were males, mean age of the patients: 57.04 years± SD 10.88 for group I and 58.80 years ± SD 11.58 for group II]. The therapy results showed a significant improvement from the baseline in both groups. There was no significant difference in the post-therapy results between group I and group II except for some items whereas group I showed more significant improvement.

**Conclusions:**

Language therapy using a computer-based Arabic software program was as effective as the conventional therapy in the improvement of language abilities of Arabic-speaking aphasic patients.

## Background

Aphasia is considered an acquired communication disorder. It affects persons who had previously learned and properly used language for communication [1]. Stroke is the most common cause of brain injury leading to aphasia. The percentage of aphasia in acute stroke patients varies from 14 to 38% [2]. In patients with aphasia, all language modalities may be disturbed like speaking, understanding, writing, and reading. There are several types of aphasia as Broca’s Aphasia in which language output is impaired with relative preservation of comprehension and Wernicke’s Aphasia which has impaired comprehension with fluent but meaningless spontaneous speech. Aphasia includes other types as global, conduction, anomic aphasia, and transcortical aphasia (motor, sensory, and mixed types) [3].

The goal of language therapy in aphasia is to help the individual to attain the highest level of independent function and involvement in daily living. The main target of language therapy is the improvement of communication and the quality of life [4]. Language intervention in aphasia helps in optimizing the patient outcomes [5].

The goal and course of rehabilitation should be determined according to the patient, the caregivers/family, and other healthcare professionals. Cultural background, language preference, job, and individual interests should not be ignored [6]. Aphasia intervention depends heavily on conventional methods of language therapy delivered by a qualified phoniatrician with direct patient care. Lack of immediate access to rehabilitation centers and deficiency of phoniatricians can lead to the limited effectiveness of the conventional language therapy. Prolonged language therapy is also needed due to the slow recovery of language functions following any stroke, leading to a heavy financial load on the caregivers.

Recently Computer programs are developed for the treatment of aphasia. These programs provide exercises that can be carried out regularly targeting vocabulary and concentrating on the patient’s conversational needs. Such software programs are valuable in the delivery of intensive independent language practice. They offer opportunities for self-management of continued aphasia treatment [7, 8]. Computer-based aphasia treatment in the long term is expected to be an effective and a low-cost choice [9, 10]. It offers a cost-effective treatment suitable for people with limited resources [11, 12].

There are a lot of aphasia software programs available in various languages. It is necessary to develop software programs for aphasia rehabilitation in the Arabic language to help Arabic-speaking patients in improving their language skills and quality of life. Arabic software programs can be an alternative to the conventional language therapy for those patients. There was an Arabic software program developed several years ago for aphasia rehabilitation, but it did not target all language defects of aphasia. Perseveration, reading, writing, and arithmetic defects need an adequate rehabilitation that was not adequately handled in the previous software [13].

Therefore, our objective in the present study was to develop a software program in Arabic language that can deal with the majority of language defects of aphasia and determine its effectiveness. The effectiveness of this software in improving language skills of post-stroke aphasic patients was assessed when given by a phoniatrician, in comparison to the conventional language therapy. The results of this study could provide important insights on whether future studies could be done to assess if the computer-based language rehabilitation therapy for aphasia delivered by the caregivers at home can be offered as a cost-effective therapeutic strategy (alone or combined with the conventional therapy) especially in resource-limited settings and presence of physical disabilities. The intervention was determined to be performed by a phoniatrician to guard against poor compliance of the patients and caregivers in using the therapy program at home. Phoniatrician ensured proper use of the program according to the language defects and disease severity. Any problems during therapy could be managed and acceptability of the patients to this type of intervention could be improved. Phoniatrician-based therapy can overcome the problem of limited computer resources and poor computer skills. We hope that all these obstacles will be resolved in the near future permitting the use of computer-based software therapy by aphasic patients and their caregivers at home.

## Methods

### Study design

Patients with aphasia who attended the Phoniatrics unit, Otorhinolaryngology Department from January 2018 to September 2019 were enrolled in a randomized controlled trial with blinded endpoint evaluation. The aim was to develop a software program in the Arabic language that can deal with the majority of language defects of aphasia and compare the effect of language therapy using this software program versus the conventional language therapy to test its effectiveness. The study was approved by the Ethics Committee, Faculty of Medicine.

### Study population

The eligibility of 105 aphasic patients who attended the Phoniatrics unit was assessed. Fifty subjects met the eligibility criteria. We included post-stroke aphasic patients who had 18 years old or more. All included patients were Arabic speaking. Patients had aphasia in any phase (acute- subacute-chronic). The acute phase represents the first 3 months after the occurrence of aphasia. The subacute phase represents 3–6 months after the occurrence of aphasia, while the chronic phase represents more than 6 months after the onset of aphasia.

We excluded patients who had intellectual disabilities, visual impairment, hearing impairment, associated dysarthria, and or apraxia of speech and associated psychiatric disorders. Patients were excluded based on clinical examination, medical reports, and assessment of intellectual abilities, hearing, and vision. All patients with acceptable visual and hearing thresholds were included.

The research steps and benefits were explained to the patients and caregivers. We answered any questions and provided any needed explanation. All participants or their caregivers provided a written consent before starting the intervention.

### Study steps

The study included several steps which were:

#### Step 1

Designing the software program was done by a software designing company. The software is called “Rawag.” During the developmental stage of the software, there were periodic assessments and modifications of the program if necessary. This software included 10 sections to be a more detailed and comprehensive program that covered most of the linguistic deficits in aphasic patients as reading, writing, and mathematical deficits. Every section was organized in a hierarchy according to task difficulty and language complexity. The software program sections are shown in Table 1.

**Table 1:** The software program sections

Program sections	Items of each section
1- Auditory comprehension training materials	▪ Yes or no questions. ▪ Orders. ▪ Picture matching.
2- Word-finding training materials	▪ Picture naming (the program includes a lot of pictures to represent variable semantic categories as body parts, fruits, vegetables, animals, birds, insects, transportation, kitchen tools, common objects, plants, flowers, furniture, clothes, occupations, colors, geometric figures, verbs, and picture description) ▪ Choose the correct semantic category ▪ Choose the correct answer that belongs to each semantic category. ▪ Mention the category name. ▪ Choose the word that does not belong to each semantic category. ▪ Mention the name of the object. ▪ Mention the characteristics of each word. ▪ Complete (include several parts as associations, opposites, prepositions, and verbs) ▪ List types of each group. ▪ Choose the correct answer.
3- Sentence structure exercises	▪ Reading or repetition of words, phrases, and sentences. ▪ Rearrange the words to make a sentence.
4- Oral expression training materials	▪ Mention ways the paired items are alike. ▪ Description of different tasks. ▪ Describe what these familiar sayings mean.
5- Writing	▪ Writing letters. ▪ Matching letters. ▪ Writing the picture name.
6- Spelling	▪ Choose the word that is spelled correctly. ▪ Write the missing letter to complete each word.
7- Reading	▪ Read the statement and choose the correct answer. ▪ Mention the missing words.
8- Arithmetic	▪ Add. ▪ Subtract. ▪ Multiply. ▪ Divide. ▪ Complete the following sentences. ▪ Answer the following questions.
9- Time	▪ Read the time shown on the clock and write it. ▪ Draw hands on the clock to indicate the time shown.
10- Perseveration treatment	▪ Picture naming using different methods of cues. It includes pictures of body parts, common objects, colors, numbers, letters, verbs, and figures

All the training materials were designed as a software program in the Arabic language in a detailed, easy way using the available progress in computer technology. The core concept of the program depends on the principles of Schuell’s Stimulation Approach which depends on intensive auditory stimulation to facilitate language recovery. This approach involves several principles as using repetitive sensory stimulation to elicit the maximum number of responses, intensive, and systematic work with feedback[14].

**Fig. 1:**
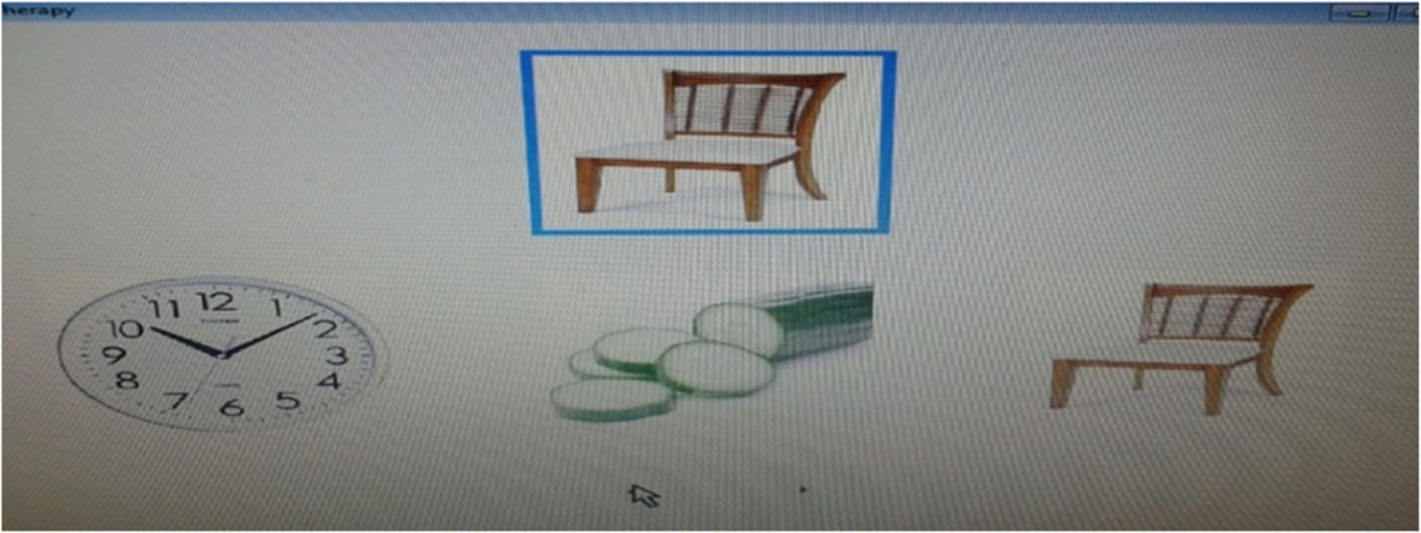
Picture matching window in the auditory comprehension section

Patients can record their answers and replay them again to recognize their mistakes. The program provides visual and or auditory feedback after getting the answers. In visual feedback, the correct answer appears green and the wrong answer appears red as in word-finding, spelling, and reading sections. Visual feedback can be also in the form of right or wrong marks as in the writing section. The program is provided with model answers to the questions in every section. These answers can be verbal, written, or both according to the question. All these can provide some independence if the patient wants to use the software alone at home. The program can be administered using a laptop or desktop computer. The software program contains easily handled large buttons suitable for aphasic patients with physical and medical comorbidities. The software program is valuable in the delivery of intensive and continued language therapy. It provides a large number of stimuli and variable types of questions targeting various language defects. Word-finding section includes a large number of pictures which represent different semantic categories to enable more practice during therapy. This software helps the phoniatrician by providing large scale of organized language exercises that target different aphasia severity even the mild form. The program has a specific section that can help the patient to read the time and write it. This step has a great effect on his quality of life and helps the patient to return to his daily life activities. The auditory comprehension window is shown in Fig. 1.

#### Step 2

Validation of the software program was performed by 5 phoniatricians who judged the program pictures and questions as a valid material. They ascertain that the program materials were suitable for aphasic patients with different disease severity. They reviewed the program sections and confirmed the suitability of all language materials. All words and sentences were suitable for Arabic-speaking patients. Any confusing or misleading phrases and sentences were changed.

#### Step 3

All eligible patients were subjected to examination in the form of complete history taking, complete neurological examination with imaging studies (brain CT and, or MRI to document the cause and the site of the stroke), and language evaluation. Both informal and formal language assessment was done to determine the type of aphasia according to the Boston classification. The formal language assessment was performed by a phoniatrician using the Arabic version of the Boston Diagnostic Aphasia Examination (BDAE) as a baseline assessment (pre-therapy). Blinded evaluation by a phoniatrician was done pre- and post-therapy.

Eligible patients were randomly assigned to receive language therapy using either the Arabic software program (group I) or the conventional therapy (group II). Conventional therapy is explained in Table 2. Both groups received therapy in the form of 2 sessions weekly. The duration of each session was 60 min. The therapy was conducted for 48 sessions over 6 months. No group therapy was done. The intervention was done by the same phoniatrician for both groups. The phoniatrician helped illiterate patients and those with minimal computers skills during sessions. The phoniatrician taught patients and caregivers how to deal with the program to facilitate more revision at home after the session; thus, family members could help the patient at home. The program is clear and easy to use even by people with minimal computer skills. We determined 90% response rate to move from one step to another in therapy for both groups. Patients received therapy in the Phoniatrics unit. All patients showed good compliance with no dropout in both groups and all randomized patients completed the trial. All patients were reassessed using the same protocol after therapy.

**Table 2:** The conventional therapy items

▪ Training materials for the improvement of receptive language	▪ One word level (picture recognition, yes or no questions, choosing the correct answer) ▪ Two-word level (yes or no questions, orders, choosing the correct tool or picture) ▪ Three-word level (orders using body parts, pictures, and real objects—pointing to the pictures in the same order as mentioned—yes or no questions) ▪ More than three-word level (orders using body parts or visual stimuli or time relationship—yes or no questions)
▪ Training materials for the improvement of expressive language	▪ One-word level (repetition of monosyllabic then multisyllabic words—completing the sentences with nouns or verbs with or without visual aids—answering questions with or without visual or auditory aids for practicing different semantic categories) ▪ Two- or three-word level (mention objects that belong to each semantic group—answering questions about object function—answering questions with or without auditory aids—completing sentences)
▪ Training materials for the improvement of reading and writing	▪ Reading words then arranging words to make a sentence. ▪ Writing words. ▪ Writing the meaning of these words. ▪ Writing the opposite of these words. ▪ Writing the missing word to complete these sentences. ▪ Choosing the correct answer. ▪ Arranging phrases to make a story. ▪ Answering questions.

#### Randomization

An independent statistician generated a manual of unique numbers to determine the study intervention allocated to each randomized patient.

**Fig. 2:**
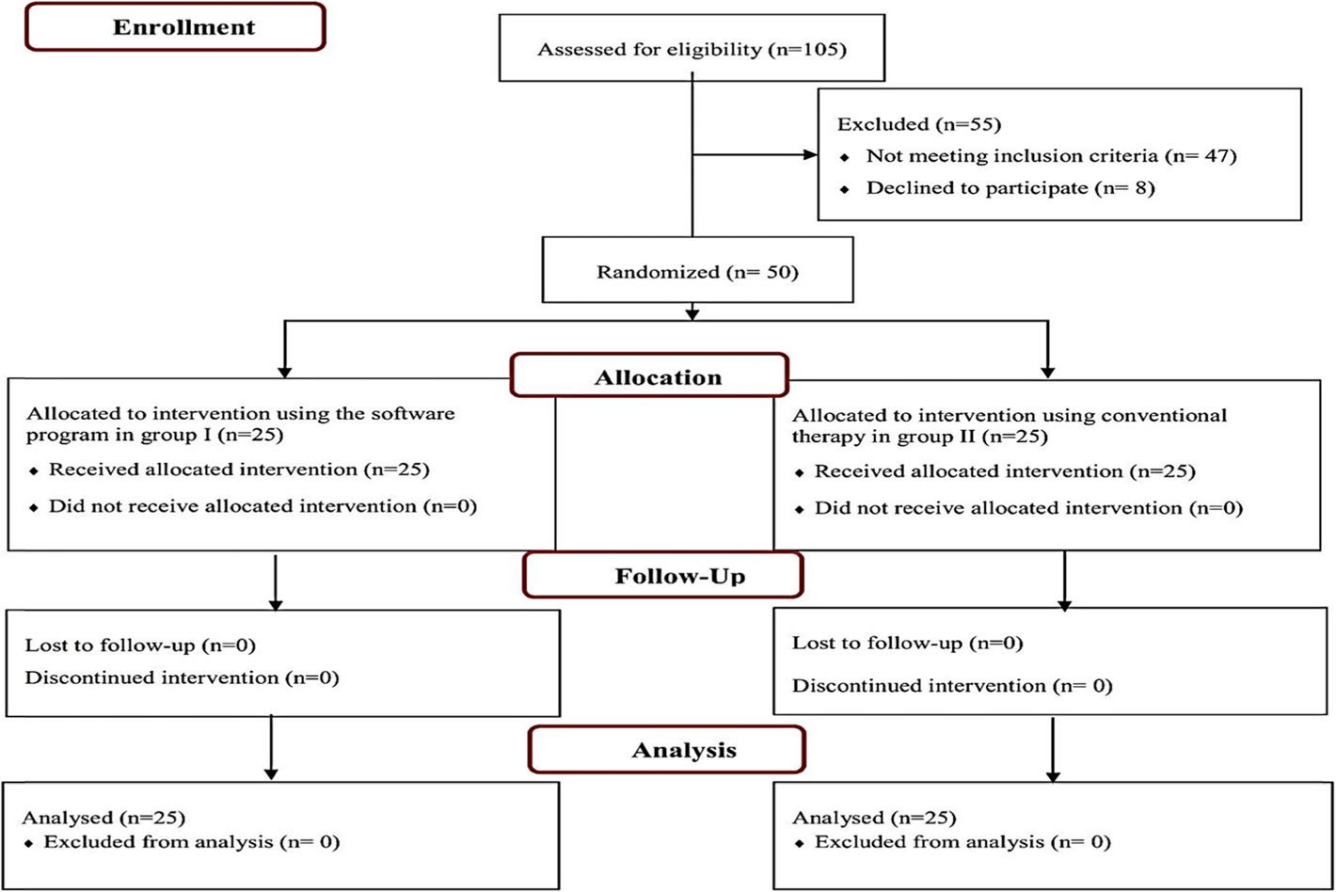
Flow diagram of the study

The statistician randomly assigned each number to the treatment groups using a simple randomization technique with the help of a random number generator program. The statistician kept this allocation concealed, and no one in the research team knew it till the patient’s assignment. The clinician who determined if a subject was eligible for inclusion in the trial was also unaware of which group the subject would be allocated. The flow diagram of the study is shown in Fig. 2.

### Study outcomes

The primary outcome was a measure of improvement in language abilities of the patients using the Arabic version of the Boston Diagnostic Aphasia Examination (BDAE). The primary outcome was measured by a blinded independent phoniatrician after 48 therapy sessions over 6 months. The results of BDAE done pre-therapy were compared to the post-therapy results to detect any significant improvement in the language of each group, and the post-therapy results of both groups were compared to detect any significant difference between them. BDAE is a battery designed to evaluate adult patients suspected of having aphasia. It evaluates different language skills as perceptual skills (auditory, visual, and gestural), processing functions (comprehension and analysis), and response skills (writing and articulation). It has five subtests (conversational and expository speech, auditory comprehension, oral expression, reading, and writing).

### Sample size and statistical analysis

The sample size was estimated to be 50 patients to detect differences between the two groups. Data were analyzed using IBM SPSS software package version 20.0*.* (Armonk, NY: IBM Corp) with α of 0.05. The significance of the results was judged at the 5% level.

We used chi-square test to compare between the two groups (for categorical variables as the demographic data). Mann-Whitney test was used to compare between the two groups (for BDAE parts), and Wilcoxon signed ranks test was used for comparing between pre and post-therapy results in each group.

## Results

### Study population

A total of 105 aphasic patients were screened for eligibility and 50 subjects met the eligibility criteria. Eligible patients were randomized to receive language therapy using either the Arabic software program (group I) or the conventional therapy (group II). All randomized patients completed the trial. The demographic data of the patients are demonstrated in Table 3. As regards sex distribution, the sample was 80% males and 20% females in both groups.

**Table 3:** Distribution of the study population according to different demographic data

Variables	Group I	Group II
Number of patients	25	25
Age (in years)		
Mean (SD)	57.04±10.88	58.80±11.58
Sex		
Males	80%	80%
Females	20%	20%
Education		
Illiterate	20%	24%
Low educational level	8%	16%
Middle educational level	36%	28%
High educational level	36%	32%
Cause of stroke		
Ischemic stroke	96%	100%
Hemorrhagic stroke	4%	0.0%
Type of aphasia		
Global aphasia	32%	32%
Broca’s aphasia	28%	20%
Transcortical motor aphasia	16%	20%
Transcortical mixed aphasia	20%	28%
Anomic aphasia	4%	0.0%
Phases of aphasia		
Acute	8%	8%
Subacute	0%	4%
Chronic	92%	88%

Group I received computer-based software therapy, group II received the conventional therapy.

Low educational level: primary education, middle educational level: secondary education, high educational level: university education

Acute phase (within 3 months after aphasia occurrence). Subacute phase (3–6 months after aphasia occurrence). Chronic phase (6 months after aphasia occurrence)

As regards age distribution, the mean age was 57.04±10.88 years in group I and 58.80±11.58 years in group II.

Radiological finding (CT and, or MRI) of all patients in both groups showed a stroke in the middle cerebral artery distribution. All patients had ischemic stroke except 4% of patients in group I had a hemorrhagic stroke. All the patients were right-handed. As regards education, five patients were illiterate in group I while the illiterate patients were six in group II.

Global aphasia represented the most common type of aphasia in the study, it accounted for 32% in both groups. Broca’s aphasia represented 28% in group I and 20% in group II. Patients with Wernicke’s and conduction aphasia were found among the screened subjects, but they were not eligible for the study. Most of the patients were in the chronic phase of aphasia (6 months after the onset of aphasia).

### Boston diagnostic aphasia examination results

#### Conversational and expository speech part

Tables 4 and 5 show the results of the conversational and expository speech part of BDAE. Both groups showed a significant improvement post-therapy in all items except word-finding relative to fluency item in group II (p value was insignificant, p = 0.179). There was an insignificant difference between both groups in the post-therapy results of all items (p > 0.05) except the Melodic line item, while the mean differences of pre- and post-therapy results between both groups were significant for some items (phrase length, melodic line, word-finding relative to fluency, and paraphasia) in favor of group I.

**Table 4:** Results of conversational and expository speech part of BDAE: simple social responses and the Aphasia severity rating scale

	Conversational and expository speech	Group I (n=25)	Group II (n=25)	p
Simple social responses	**Pre-therapy**			
	Min.–Max.	0.0–6.0	0.0–4.0	0.705
	Mean ± SD.	1.96 ± 1.46	1.76±1.48	
	**Post-therapy**			
	Min.–Max.	2.0–7.0	2.0–7.0	**0.264**
	Mean ± SD.	5.08 ± 1.58	4.60 ± 1.47	
	**The mean difference** Mean ± SD.	3.12 ± 1.01	2.84 ± 0.80	**0.250**
	**p**_**1**_	<0.001^*^	<0.001^*^	
Aphasia severity rating scale	**Pre-therapy**			
	Min.–Max.	0.0–2.0	0.0–2.0	0.598
	Mean ± SD.	0.80 ± 0.58	0.92 ± 0.76	
	**Post- therapy**			
	Min.–Max.	2.0–5.0	1.0–5.0	**0.708**
	Mean ± SD.	3.28 ± 1.02	3.36±1.08	
	**The mean difference** Mean ± SD.	2.48 ± 0.77	2.44 ± 0.92	**0.975**
	**p**_**1**_	<0.001^*^	<0.001^*^	

Group I received computer-based software therapy, group II received the conventional therapy

P: p value for comparing between the two studied groups. p_1_: p value for comparing between pre and post-therapy in each group. *P value is statistically significant when p value ≤ 0.05

**Table 5:** Results of conversational and expository speech part of BDAE: the rating scale profile of speech characteristics

	Rating scale profile of speech characteristics	Group I (n=25)	Group II (n=25)	p
Articulatory agility	**Pre-therapy**			
	Min.–Max.	1.0–7.0	1.0–7.0	0.177
	Mean ± SD.	5.28 ± 1.17	5.48 ± 1.69	
	**Post-therapy**			
	Min.–Max.	5.0–7.0	4.0–7.0	**0.478**
	Mean ± SD.	6.52 ± 0.59	6.52 ± 0.87	
	**The mean difference** Mean ± SD.	1.24 ± 0.78	1.04 ± 1.14	**0.132**
	**p**_**1**_	<0.001^*^	<0.001^*^	
Phrase length	**Pre-therapy**			
	Min.–Max.	1.0–4.0	1.0–4.0	0.181
	Mean ± SD.	1.44 ± 0.82	1.84 ± 1.11	
	**Post-therapy**			
	Min.–Max.	2.0–7.0	2.0–6.0	**0.565**
	Mean ± SD.	4.24 ± 1.64	3.96 ± 1.37	
	**The mean difference** Mean ± SD.	2.80 ± 1.22	2.12 ± 0.73	0.036^*^
	**p**_**1**_	<0.001^*^	<0.001^*^	
Grammatical forms	**Pre-therapy**			
	Min.–Max.	1.0–4.0	1.0–3.0	0.148
	Mean ± SD.	1.16 ± 0.62	1.28 ± 0.54	
	**Post-therapy**			
	Min.–Max.	2.0–7.0	2.0–5.0	**0.400**
	Mean ± SD.	4.04 ± 1.34	3.68 ± 1.11	
	**The mean difference** Mean ± SD.	2.88 ± 1.17	2.40 ± 1.04	**0.174**
	**p**_**1**_	<0.001^*^	<0.001^*^	
Melodic line	**Pre-therapy**			
	Min.–Max.	1.0–5.0	1.0–5.0	0.786
	Mean ± SD.	2.52 ± 0.82	2.40±1.08	
	**Post-therapy**			
	Min.–Max.	3.0–7.0	2.0–7.0	0.020^*^
	Mean ± SD.	5.56±1.12	4.44±1.64	
	**The mean difference** Mean ± SD.	3.04 ± 1.06	2.04 ± 1.06	0.002^*^
	**p**_**1**_	<0.001^*^	<0.001^*^	
Word-finding relative to fluency	**Pre-therapy**			
	Min.–Max.	3.0–7.0	1.0–7.0	0.025*
	Mean ± SD.	4.36±0.95	5.12±1.33	
	**Post-therapy**			
	Min.–Max.	4.0–6.0	3.0–7.0	**0.842**
	Mean ± SD.	5.64±0.57	5.60±0.82	
	**The mean difference** Mean ± SD.	1.28 ± 1.10	0.48 ± 1.73	0.003^*^
	**P**_**1**_	<0.001^*^	0.179	
Paraphasia	**Pre-therapy**			
	Min.–Max	1.0–7.0	1.0–7.0	0.518
	Mean ± SD.	2.08±2.0	2.72±2.34	
	**Post-therapy**			
	Min.–Max.	1.0–7.0	1.0–7.0	**0.275**
	Mean ± SD.	6.0±1.58	5.0±2.40	
	**The mean difference** Mean ± SD.	3.92±2.25	2.28±2.01	0.015^*^
	**P**_**1**_	<0.001^*^	<0.001^*^	

Group I received computer-based software therapy, group II received the conventional therapy

P: p value for comparing between the two studied groups. p_1_: p value for comparing between pre and post-therapy in each group. *P value is statistically significant when p value ≤ 0.05

### Auditory comprehension part

The results of the auditory comprehension part of BDAE are shown in Table 6. There was a significant improvement in both groups (p value was significant, p ≤ 0.05). We found an insignificant difference in the post-therapy results between group I and group II (p > 0.05). The mean differences of pre and post-therapy results between both groups were insignificant.

**Table 6:** Results of auditory comprehension part of BDAE

	Auditory comprehension	Group I (n=25)	Group II (n=25)	P
Basic word discrimination	**Pre-therapy**			
	Min.–Max.	2.0–37.0	2.0–36.0	0.448
	Mean ± SD.	21.40 ± 13.14	19.52 ± 12.94	
	**Post-therapy**			
	Min.–Max.	23.0–37.0	11.0–36.0	**0.467**
	Mean ± SD.	31.96 ± 4.69	29.88 ± 7.49	
	**The mean difference** Mean ± SD.	10.56 ± 9.22	10.36 ± 8.53	**0.891**
	**p**_**1**_	<0.001^*^	<0.001^*^	
Commands	**Pre-therapy**			
	Min.–Max.	0.0–15.0	0.0–15.0	0.597
	Mean ± SD.	7.08 ± 5.73	6.64 ± 5.69	
	**Post-therapy**			
	Min.–Max.	7.0–15.0	5.0–15.0	**0.595**
	Mean ± SD.	11.96 ± 2.49	11.44 ± 3.07	
	**The mean difference** Mean ± SD.	4.88 ± 3.68	4.80 ± 3.81	**0.876**
	**p**_**1**_	<0.001^*^	<0.001^*^	
Complex ideational material	**Pre-therapy**			
	Min.–Max.	0.0–11.0	0.0–10.0	0.491
	Mean ± SD.	4.52 ± 3.96	3.96 ± 4.41	
	**Post-therapy**			
	Min.–Max.	4.0–12.0	4.0–11.0	**0.224**
	Mean ± SD.	9.12 ± 2.32	8.36 ± 2.36	
	**The mean difference** Mean ± SD.	4.60 ± 2.27	4.40 ± 2.97	**0.703**
	**p**_**1**_	<0.001^*^	<0.001^*^	

Group I received computer-based software therapy, group II received the conventional therapy

P: p value for comparing between the two studied groups. p_1_: p value for comparing between pre and post-therapy in each group. *P value is statistically significant when p value ≤ 0.05

### Oral expression part

Tables 7, 8, 9, and 10 show the results of the oral expression part. There was a significant improvement in all items of oral expression part after therapy in both groups (p value was significant, p ≤ 0.05). The mean differences of pre and post-therapy results between both groups were significant for some items (responsive naming, Boston naming test, and repetition item) where group I showed more significant improvement.

**Table 7:** Results of oral expression part of BDAE: oral agility item

	Oral agility	Group I (n=25)	Group II (n=25)	P
Nonverbal agility	**Pre-therapy**			
	Min.–Max.	0.0–10.0	0.0–7.0	0.731
	Mean ± SD.	3.56 ± 2.29	3.24 ± 2.01	
	**Post-therapy**			
	Min.–Max.	6.0–12.0	5.0–11.0	**0.070**
	Mean ± SD.	9.08 ± 2.0	8.12 ± 1.96	
	**The mean difference** Mean ± SD.	5.52 ± 1.78	4.88 ± 2.15	**0.250**
	**p**_**1**_	<0.001^*^	<0.001^*^	
Verbal agility	**Pre-therapy**			
	Min.–Max.	0.0–12.0	0.0–7.0	0.758
	Mean ± SD.	2.76 ± 2.39	2.40 ± 1.78	
	**Post-therapy**			
	Min.–Max.	6.0–14.0	5.0–12.0	**0.056**
	Mean ± SD.	10.36 ± 2.58	9.12 ± 2.35	
	**The mean difference** Mean ± SD.	7.60 ± 2.55	6.72 ± 2.30	**0.264**
	**p**_**2**_	<0.001^*^	<0.001^*^	

Group I received computer-based software therapy, group II received the conventional therapy

P: p value for comparing between the two studied groups. p_1_: p value for comparing between pre and post-therapy in each group. *P value is statistically significant when p value ≤ 0.05

**Table 8:** Results of oral expression part of BDAE: automatized sequences item and recitation, melody, and rhythm item

		Group I (n=25)	Group II (n=25)	P
Automatized sequences	**Pre-therapy**			
	Min.–Max.	0.0–8.0	0.0–6.0	0.574
	Mean ± SD.	1.92 ± 1.63	2.0 ± 1.35	
	**Post-therapy**			
	Min.–Max.	2.0–8.0	2.0–8.0	**0.570**
	Mean ± SD.	6.16 ± 1.70	5.96 ± 1.43	
	**The mean difference** Mean ± SD.	4.24 ± 1.51	3.96 ± 1.27	**0.359**
	**p**_**1**_	<0.001^*^	<0.001^*^	
Recitation, melody, and rhythm	**Pre-therapy**			
	Min.–Max.	0.0–4.0	0.0–3.0	0.492
	Mean ± SD.	1.68 ± 1.22	1.76 ± 0.88	
	**Post-therapy**			
	Min.–Max.	2.0–6.0	2.0–5.0	**0.302**
	Mean ± SD.	4.40 ± 1.32	4.0 ± 0.91	
	**The mean difference** Mean ± SD.	2.72 ± 0.94	2.24 ± 0.88	**0.052**
	**p**_**1**_	<0.001^*^	<0.001^*^	

Group I received computer-based software therapy, group II received the conventional therapy

P: p value for comparing between the two studied groups. p_1_: p value for comparing between pre and post-therapy in each group. *P value is statistically significant when p value ≤ 0.05

**Table 9:** Results of oral expression part of BDAE: repetition item

	Repetition	Group I (n=25)	Group II (n=25)	p
Single words	**Pre-therapy**			
	Min.–Max.	0.0–10.0	0.0–10.0	0.550
	Mean ± SD.	3.72 ± 3.20	4.36 ± 3.47	
	**Post-therapy**			
	Min.–Max.	4.0–10.0	4.0–10.0	**0.454**
	Mean ± SD.	7.72 ± 1.90	7.28 ± 2.15	
	**The mean difference** Mean ± SD.	4.0 ± 1.94	2.92 ± 1.80	0.040^*^
	**p**_**1**_	<0.001^*^	<0.001^*^	
Sentence	**Pre-therapy**			
	Min.–Max.	0.0–9.0	0.0–9.0	0.724
	Mean ± SD.	2.44 ± 2.89	2.48 ± 2.93	
	**Post-therapy**			
	Min.–Max.	4.0–10.0	3.0–10.0	**0.128**
	Mean ± SD.	6.52 ± 1.98	5.72 ± 2.39	
	**The mean difference** Mean ± SD.	4.08 ± 1.47	3.24 ± 1.27	0.049^*^
	**p**_**1**_	<0.001^*^	<0.001^*^	

Group I received computer-based software therapy, group II received the conventional therapy

P: p value for comparing between the two studied groups. p_1_: p value for comparing between pre and post-therapy in each group. *P value is statistically significant when p value ≤ 0.05

**Table 10:** Results of oral expression part of BDAE: naming item

	Naming	Group I (n=25)	Group II (n=25)	p
Responsive naming	**Pre-therapy**			
	Min.–Max.	0.0–12.0	0.0–17.0	0.686
	Mean ± SD.	2.56 ± 2.50	3.12 ± 3.62	
	**Post-therapy**			
	Min.–Max.	9.0–20.0	5.0–20.0	0.021^*^
	Mean ± SD.	15.44 ± 3.82	12.60 ± 4.09	
	**The mean difference** Mean ± SD.	12.88 ± 2.93	9.48 ± 3.51	0.001*
	**p**_**1**_	<0.001^*^	<0.001^*^	
Boston naming test	**Pre-therapy**			
	Min.–max.	0.0–46.0	0.0–25.0	0.770
	Mean ± SD.	7.92 ± 9.22	6.92 ± 6.38	
	**Post-therapy**			
	Min.–Max.	26.0–60.0	12.0–54.0	0.003^*^
	Mean ± SD.	47.04 ± 11.06	37.08 ± 11.33	
	**The mean difference** Mean ± SD.	39.12 ± 10.15	30.16 ± 10.55	0.003*
	**p**_**1**_	<0.001^*^	<0.001^*^	
Screening of special categories	**Pre-therapy**			
	Min.–Max.	0.0–9.0	0.0–9.0	0.315
	Mean ± SD.	3.76 ± 2.37	3.04 ± 2.26	
	**Post-therapy**			
	Min.–Max.	6.0–12.0	4.0–12.0	**0.080**
	Mean ± SD.	9.92 ± 1.91	8.88 ± 2.28	
	**The mean difference** Mean ± SD.	6.16 ± 1.62	5.84 ± 2.34	**0.477**
	**p**_**1**_	<0.001^*^	<0.001^*^	

Group I received computer-based software therapy, group II received the conventional therapy

P: p value for comparing between the two studied groups. p_1_: p value for comparing between pre and post-therapy in each group. *P value is statistically significant when p value ≤ 0.05

### Reading part

Tables 11, 12, 13, and 14 show the results of the reading part of BDAE whereas most items had a significant improvement after therapy in both groups (the p value was significant ≤ 0.05) except in matching item in group I; there was an insignificant improvement (p value was insignificant, p = 0.083). The mean differences of pre and post-therapy results between both groups were insignificant for all items except matching item where group II showed more significant improvement.

**Table 11:** Results of reading part of BDAE: basic symbol recognition

	Basic symbol recognition	Group I (n=20)	Group II (n=19)	P
Matching	**Pre-therapy**			
	Min.–Max.	6.0–8.0	0.0–8.0	0.033^*^
	Mean ± SD.	7.75 ± 0.55	6.37 ± 2.63	
	**Post-therapy**			
	Min.–Max.	6.0–8.0	3.0–8.0	**0.550**
	Mean ± SD.	7.90 ± 0.45	7.32 ± 1.63	
	**The mean difference** Mean ± SD.	0.15 ± 0.37	0.95 ± 1.22	0.015*
	**p**_**1**_	**0.083**	0.002^*^	
Number matching	**Pre-therapy**			
	Min.–Max.	9.0–12.0	0.0–12.0	0.967
	Mean ± SD.	11.15 ± 0.99	10.11 ± 3.56	
	**Post-therapy**			
	Min.–Max.	10.0–12.0	5.0–12.0	**0.792**
	Mean ± SD.	11.70 ± 0.57	11.05 ± 2.27	
	**The mean difference** Mean ± SD.	0.55 ± 0.60	0.95 ± 1.39	**0.667**
	**p**_**1**_	0.002^*^	0.004^*^	

Group I received computer-based software therapy, group II received the conventional therapy

P: p value for comparing between the two studied groups. p_1_: p value for comparing between pre and post-therapy in each group. *P value is statistically significant when p value ≤ 0.05

**Table 12:** Results of reading part of BDAE: word identification

	Word identification	Group I (n=20)	Group II (n=19)	P
Picture word match	**Pre-therapy**			
	Min.–Max.	0.0–10.0	0.0–10.0	0.166
	Mean ± SD.	8.35 ± 2.35	7.58 ± 2.83	
	**Post-therapy**			
	Min.–Max.	4.0–10.0	4.0–10.0	**0.175**
	Mean ± SD.	9.40 ± 1.57	9.0 ± 1.70	
	**The mean difference** Mean ± SD.	1.05 ± 0.94	1.42 ± 1.35	**0.478**
	**p**_**1**_	<0.001^*^	<0.001^*^	
Lexical decision	**Pre-therapy**			
	Min.–Max.	0.0–5.0	0.0–10.0	0.270
	Mean ± SD.	4.0 ± 1.08	3.84 ± 2.52	
	**Post-therapy**			
	Min.–Max.	2.0–5.0	2.0–10.0	**0.336**
	Mean ± SD.	4.75 ± 0.72	4.79 ± 2.12	
	**The mean difference** Mean ± SD.	0.75 ± 0.64	0.95 ± 0.62	**0.396**
	**p**_**1**_	0.001^*^	<0.001^*^	

Group I received computer-based software therapy, group II received the conventional therapy

P: p value for comparing between the two studied groups. p_1_: p value for comparing between pre and post-therapy in each group. *P value is statistically significant when p value ≤ 0.05

**Table 13:** Results of reading part of BDAE: homophone matching and matching to spoken sample

		Group I (n=20)	Group II (n=19)	P
Homophone matching	**Pre-therapy**			
	Min.–Max.	0.0–5.0	0.0–4.0	0.175
	Mean ± SD.	3.45 ± 1.32	3.0 ± 1.41	
	**Post-therapy**			
	Min.–Max.	2.0–5.0	2.0–5.0	0.005^*^
	Mean ± SD.	4.50 ± 0.83	3.79 ± 0.85	
	**The mean difference** Mean ± SD.	1.05 ± .83	0.79 ± 0.79	**0.380**
	**p**_**1**_	<0.001^*^	0.002^*^	
Matching to spoken sample	**Pre-therapy**			
	Min.–Max.	0.0–10.0	0.0–10.0	0.513
	Mean ± SD.	6.85 ± 2.92	6.63 ± 2.77	
	**Post-therapy**			
	Min.–Max.	3.0–10.0	3.0–10.0	**0.396**
	Mean ± SD.	8.45 ± 1.90	8.16 ± 1.89	
	**The mean difference** Mean ± SD.	1.60± 1.19	1.53 ± 1.26	**0.835**
	**p**_**1**_	<0.001^*^	<0.001^*^	

Group I received computer-based software therapy, group II received the conventional therapy.

P: p value for comparing between the two studied groups. p_1_: p value for comparing between pre and post-therapy in each group. *P value is statistically significant when p value ≤ 0.05

**Table 14:** Results of reading part of BDAE: basic oral reading, oral reading of sentence with comprehension, and reading comprehension items

		Group I (n=20)	Group II (n=19)	p
Basic oral reading	**Pre-therapy**			
	Min.–Max.	0.0–30.0	0.0–20.0	0.184
	Mean ± SD.	6.15 ± 8.45	3.95 ± 5.53	
	**Post-therapy**			
	Min.–Max.	4.0–30.0	3.0–26.0	**0.380**
	Mean ± SD.	16.45 ± 7.41	14.16 ± 7.08	
	**The mean difference** Mean ± SD.	10.30 ± 5.32	10.21± 6.24	**0.945**
	**p**_**1**_	<0.001^*^	<0.001^*^	
Oral reading of sentences with comprehension	**Pre-therapy**			
	Min.–Max.	0.0–13.0	0.0–11.0	0.044^*^
	Mean ± SD.	4.85 ± 2.87	3.21 ± 2.76	
	**Post-therapy**			
	Min.–Max.	5.0–15.0	4.0–12.0	**0.061**
	Mean ± SD.	9.45 ± 2.86	7.74 ± 2.49	
	**The mean difference** Mean ± SD.	4.60 ± 1.73	4.53± 2.04	**0.967**
	**p**_**1**_	<0.001^*^	<0.001^*^	
Reading comprehension	**Pre-therapy**			
	Min.–Max.	0.0–8.0	0.0–8.0	0.120
	Mean ± SD.	4.50 ± 2.86	3.05 ± 3.01	
	**Post-therapy**			
	Min.–Max.	3.0–10.0	3.0–9.0	**0.095**
	Mean ± SD.	7.15 ± 2.01	6.05 ± 1.65	
	**The mean difference** Mean ± SD.	2.65 ± 1.35	3.0 ± 1.70	**0.428**
	**P**_**2**_	<0.001^*^	<0.001^*^	

Group I received computer-based software therapy, group II received the conventional therapy

P: p value for comparing between the two studied groups. p_1_: p value for comparing between pre and post-therapy in each group. *P value is statistically significant when p value ≤ 0.05

### Writing part

The results of the writing part are shown in Table 15. Ten patients cannot write due to right hemiplegia with five illiterate patients in group I. Six patients cannot write due to right hemiplegia with six illiterate patients in group II. All items of the writing part showed a significant improvement after therapy in both groups (p value was significant, p ≤ 0.05.). The mean differences of pre- and post-therapy results between both groups were insignificant for all items.

**Table 15:** Results of writing part

		Group I (n=10)	Group II (n=13)	P
Mechanics of writing	**Pre-therapy**			
	Min.–Max.	2.0–60.0	15.0–60.0	0.563
	Mean ± SD.	39.70 ± 18.43	42.15 ± 16.05	
	**Post-therapy**			
	Min.–Max.	44.0–63.0	39.0–61.0	**0.410**
	Mean ± SD.	51.80 ± 6.11	53.08 ± 6.37	
	**The mean difference** Mean ± SD.	12.10 ± 16.20	10.92 ± 12.89	**0.976**
	**p**_**1**_	0.008^*^	0.001^*^	
Basic encoding skills	**Pre-therapy**			
	Min.–Max.	0.0–15.0	2.0–15.0	0.166
	Mean ± SD.	6.80 ± 5.33	9.38 ± 4.07	
	**Post-therapy**			
	Min.–Max.	6.0–16.0	3.0–16.0	**0.115**
	Mean ± SD.	10.70 ± 3.27	12.54 ± 3.48	
	**The mean difference** Mean ± SD.	3.90 ± 2.73	3.15± 3.16	**0.376**
	**P**_**2**_	0.005^*^	0.005^*^	
Written picture naming	**Pre-therapy**			
	Min.–Max.	0.0–12.0	3.0–12.0	0.343
	Mean ± SD.	6.20 ± 4.37	8.23 ± 2.77	
	**Post-therapy**			
	Min.–Max.	5.0–12.0	3.0–12.0	**0.232**
	Mean ± SD.	8.70 ± 2.67	9.92 ± 2.56	
	**The mean difference** Mean ± SD.	2.50 ± 2.27	1.69 ± 1.32	**0.522**
	**p**_**1**_	<0.001^*^	<0.001^*^	
Narrative writing	**Pre-therapy**			
	Min.–Max.	0.0–10.0	2.0–9.0	0.101
	Mean ± SD.	4.20 ± 3.08	6.46 ± 3.02	
	**Post-therapy**			
	Min.–Max.	4.0–11.0	3.0–10.0	**0.232**
	Mean ± SD.	7.10 ± 2.38	8.31 ± 2.21	
	**The mean difference** Mean ± SD.	2.90± 0.99	1.85 ± 1.52	**0.067**
	**p**_**1**_	0.005^*^	0.003^*^	

Group I received computer-based software therapy, group II received the conventional therapy

P: p value for comparing between the two studied groups. p_1_: p value for comparing between pre and post-therapy in each group. *P value is statistically significant when p value ≤ 0.05.

## Discussion

In the present study, the effectiveness of a computer-based Arabic software program in improving language skills of post-stroke aphasic patients was assessed when given by a phoniatrician, in comparison to the conventional language therapy. The mean age of the study population was 57.04±10.88 years in group I and 58.80±11.58 years in group II. It is supported by many studies that stroke incidence rises with increasing age. Aging is the strongest non-changeable risk factor for stroke. Older individuals have higher mortality, morbidity, and poorer recovery than the younger population [15, 16].

As to sex distribution, males represented 80% of the study population in both groups. It is supported by many studies that stroke is more common in males. It was attributed to the presence of many risk factors for stroke among males (as the stroke was the etiological factor of aphasia in this study) [17]. Males may seek medical help for language rehabilitation more often than females because they are most often the only breadwinner of the family, and they want to recover their language skills quickly in order to return to work.

All patients showed good compliance with no dropout in both groups and all randomized patients completed the trial. Fortunately, phoniatrics, physiotherapy, and neurology clinics are in the same building, so patients could receive all the needed medical care in the same day. This caused good compliance and prevent any dropout.

Aphasia was attributed to stroke in both groups. It was suggested to include stroke patients to avoid any confounding factors that can affect the prognosis. As in case of brain tumors which usually have a progressive course and we cannot predict the effect of any surgical or medical intervention in these cases. Stroke also represents the most common cause of aphasia.

All patients in group II had an ischemic stroke, while 96% of the patients had an ischemic stroke in group I. This is in line with what was found by numerous studies which stated that ischemic stroke is more common than hemorrhagic type [18].

As regards the type of aphasia, global aphasia represented 32% of the study population in both groups, while Broca’s aphasia was 28% in group I and 20% in group II. It is in agreement with the finding of many studies. El–Tallawy et al. 2019 found that global aphasia is the most frequent aphasia type followed by Broca’s aphasia especially in acute cases [19, 20]. Patients with more severe language impairment may tend to seek medical advice more often than the less severe impairment. This can explain the large proportion of global aphasia in the study. We could not find eligible subjects with Wernicke’s, conduction aphasia, or transcortical sensory aphasia. Chronic aphasia represented a large proportion of the patients. This is important to avoid the conflict of improvement of language abilities due to spontaneous recovery in the acute phase.

As regards the pre-therapy results of BDAE, there was an insignificant difference between both groups in most of the items. This little variation in the pre-therapy results may be due to that most of the patients who seek medical help have moderate to severe disorder so there was no great variation in the pre-therapy results.

As regards the improvement of language abilities as detected by BDAE, both groups showed significant improvement in all items after therapy except word-finding relative to fluency (conversational and expository speech part) for group II and matching item (reading part) for group I because patients had relatively good results in these items pre-therapy. The mean differences of pre and post-therapy results between both groups were insignificant for most of the items except phrase length, melodic line, word-finding relative to fluency, paraphasia, repetition, responsive naming, Boston naming test, and matching items. Group I showed more significant improvement in all these items except matching (patients had relatively good results in this item pre-therapy so there was insignificant difference post-therapy). The more significant improvement in group I might be due to the availability of varieties of pictures and exercises in the software program. This finding ensures the effectiveness of the software program as compared to the conventional therapy.

So the study concluded the effectiveness of the computer-based Arabic software program for aphasia rehabilitation. The software program was equivalent to the conventional language therapy in improvement of language skills as detected by BDAE. The software program had more superior results in some items of the test.

However, the computer-based therapy has some disadvantages as the absence of some helping strategies that are tailored for each patient according to the severity of aphasia and language skills. These strategies can be in the form of gesturing, uttering the first sound or syllable of the word, and using different cues. The computer-based therapy may lack the encouraging words related to each response and the handwriting exercises cannot be delivered. It requires a computer or a laptop and basic knowledge of using computer. A lot of these problems were managed in the present study by the presence of a phoniatrician who can tailor various helping strategies according to the defects and severity of aphasia of each patient. Phoniatrician offers a lot of support and encouragement to the patients and supply them with the needed steps to use the program at home. The handwriting exercises can be offered by the phoniatrician beside the software materials. Nowadays, we can find a computer in nearly every home in our country due to the continued efforts exerted by the government to use computer technology in all fields even in education and health. Phoniatrician ensured proper use of the program according to the language defects and disease severity. Phoniatrician-based therapy can overcome the problem of poor computer skills and motor disabilities that can hinder computer use.

The benefits of computerized software programs in the rehabilitation of aphasia had been supported by a lot of studies as that was done by Adrián, González [21]. It demonstrated that the Spanish Computer-assisted Program for anomia (CARP-2) was an active treatment for anomia. All patients showed significant benefits with carry-over in naming. Palmer, Enderby [22] studied the effectiveness of computer treatment in chronic aphasia due to stroke. This study demonstrated the early evidence of the cost-effectiveness of self-managed therapy using the computer program. The benefits of computer-based aphasia rehabilitation were confirmed by other studies as that was done by Archibald, Orange [23].

In a review to investigate the application of computer technology in Aphasiology, it was concluded that computer applications were commonly used in aphasia rehabilitation. Many of the programs for aphasia focus on disorder-oriented treatment. They give the patients a big opportunity to work individually, as often as they like. They increase the intensity of therapy. Computer programs can also add to the functional and participation goals of rehabilitation [8]. In another review done by Zheng, Lynch [24] to define the effect of aphasia rehabilitation using computer therapy, it confirmed the effectiveness of computer therapy when compared to no rehabilitation and offered primary evidence that computer-based therapy might be as effective as the therapy mediated by a clinician. This review highlighted the need for further research exploring the effect of computer therapy in a bigger sample to allow the investigation of factors as the type of aphasia, severity, the importance of feedback, and cueing effects on treatment outcome.

On the other hand, there was another study done by Kesav, Vrinda [25] to investigate the benefits of computer-based rehabilitation that reported opposite results. This study concluded that the more intensive therapy group that included combined conventional therapy and computer-based training gave inferior results than the less intensive therapy group of conventional therapy. This finding supported the significance of the conventional language therapy in enhancing the recovery of aphasia. The poor results of computer-based therapy were attributed to the poor educational level, little computer expertise, little acceptability, mental, and physical fatigue due to longer sessions.

There was a previous study to investigate the effectiveness of Arabic software program in aphasia rehabilitation. It concluded the equal benefit of both computer software and conventional therapy. This software program includes three levels (one-word sentence, two-word sentence, and three to four-word sentence level), and it did not include specific sections for reading, writing, and arithmetic rehabilitation. The present program involved many sections as reading, writing, mathematical, auditory comprehension, and perseveration training material. It is a more detailed program to address the majority of language defects in aphasia[13].

There were some limitations of the present study as patients with speech apraxia, dysarthria, and intellectual disabilities were excluded from the study. It is advisable to apply the study on those patients as aphasia is commonly associated with these disorders, and any modification can be added to the program to suit those patients. The cost-effectiveness of the computer-based aphasia rehabilitation and quality of life of patients after therapy have to be explored to document the program effect. Maintenance of the therapeutic effects of the program has to be investigated after a long time of therapy termination. It is recommended to study the effectiveness of the software program among a larger number of patients with different types and severity levels of aphasia to document its effect.

The study concluded the effectiveness of the computer-based Arabic software program for aphasia rehabilitation so it will be reassuring to apply the software program at home with the caregiver help and some follow-up visits to the clinic. The phoniatrician can train caregivers on the use of the program and supply them with the most suitable cueing strategies according to the language defects and severity. The effect of using this program at home without the clinician intervention has to be investigated to confirm its benefits as saving efforts in those older patients with physical disabilities or using it as a substitute to the conventional therapy especially in the COVID-19 pandemic. It is also important to determine the compliance of the patients and caregivers in applying this kind of therapy.

## Conclusions

The treatment group incorporating computer-based language therapy offered almost equal results as the conventional language therapy group (except for some items in which the computer-based language therapy had superior results). So it was concluded that language therapy using a computer-based Arabic software program was as effective as the conventional therapy in the improvement of language abilities of Arabic-speaking aphasic patients.

## Data Availability

The datasets used and/or analyzed during the current study are available from the corresponding author on reasonable request.

## Abbreviations

BDAE: Boston Diagnostic Aphasia Examination
CT: Computed tomography
MRI: Magnetic resonance imaging
